# Lipoic acid enhances survival of transplanted neural stem cells by reducing transplantation-associated injury

**DOI:** 10.2147/jn.s43745

**Published:** 2013-07-23

**Authors:** Junling Gao, Jason R Thonhoff, Tiffany J Dunn, Ping Wu

**Affiliations:** 1 Department of Neuroscience and Cell Biology, University of Texas Medical Branch, Galveston, TX, USA; 2 Department of Neurology, The Methodist Hospital, Houston, TX, USA

**Keywords:** neural stem cell, transplantation, hypoxia-reperfusion, antioxidant, cell survival, lipoic acid

## Abstract

The efficacy of stem cell-based therapy for neurological diseases depends highly on cell survival post-transplantation. One of the key factors affecting cell survival is the grafting procedure. The current study aims to determine whether needle insertion into intact rat spinal cords creates a hypoxic environment that is prone to lipid peroxidation damage upon reperfusion, and whether an antioxidant protects human neural stem cells (hNSCs) both in vitro and post-transplantation into rat spinal cords. We show here that a single needle injection creates a hypoxic environment within the rat spinal cord that peaks at approximately 12 hours before reperfusion occurs. Lipid peroxidation damage at the transplantation site is evident by 48 hours post-needle insertion. In an in vitro model, hypoxia-reperfusion results in apoptotic death of hNSCs. Pretreatment with the antioxidant, α-lipoic acid, protects hNSCs against hypoxia-reperfusion injury and oxidative stress–mediated cell death. Increasing glutathione, but not Akt signaling, contributes to the protective effect of lipoic acid. Pretreating hNSCs with lipoic acid also increases the cell survival rate 1 month post-transplantation. Further investigation is warranted to develop improved techniques to maximize the survival of transplanted stem cells.

## Introduction

Stem cell transplantation directly into neural tissue has the potential to treat a variety of neurological injuries and disorders. The efficacy of these treatments would depend highly on the survival rate of grafted stem cells. It has been shown that the survival rates of transplanted stem cells are variable in rodent models,^1^ and are affected by many factors. Nevertheless, how grafted cells and their surrounding environment are affected by the transplantation procedure per se has not been addressed.

The transplantation procedure may inherently pose several threats to short-term stem cell survival. For example, the transplantation needle may shear stem cells during injection and disrupt the vasculature at the transplantation site upon insertion. Grafted cells may further be affected by hypoxia-reperfusion injury caused by local vascular disruption and the subsequent burst of reactive oxygen and nitrogen species upon reperfusion. Hypoxia-reperfusion injury proceeds through a complicated mechanism involving the loss of high-energy phosphate compounds, generalized depolarization, and considerable increases in intracellular Ca^2+^ during the ischemic phase, followed by the production of oxygen radicals and nitric oxide, generating peroxynitrite and subsequent lipid peroxidation during the reperfusion stage.^2^ Along these lines, we believe that it is important to characterize hypoxia-reperfusion injury caused by the transplantation procedure. Furthermore, we hypothesize that combining stem cell injections with drug therapies aimed at counteracting several stages of the initial hypoxia-reperfusion injury immediately following transplantation may improve long-term cell survival and thus functional outcomes.

Alpha-lipoic acid (α-LA), an eight-carbon dithiol compound,^3^ is a powerful antioxidant that displays many characteristics necessary to potentially protect transplanted stem cells from hypoxia-reperfusion injury. Lipoic acid (LA) is synthesized naturally and normally acts as an essential cofactor for several dehydrogenase complexes in mitochondrial energy metabolism.^4^ LA also readily crosses the blood–brain barrier and enters cells by both passive diffusion and active uptake by a Na^+^-dependent vitamin transporter.^5^ α-LA has been used to treat diabetic and alcoholic neuropathies, is neuroprotective against glutamate-induced cytotoxicity, and protects peripheral nerves from ischemia-reperfusion injury.^6–8^ LA has also been shown to be protective against ischemia-reperfusion injury in the spinal cord^9,10^ and to reduce oxidative stress–associated damage in spinal cord injury in small animals.^11,12^ Dihydrolipoic acid (DHLA), a reduced form of R-α-LA, is a much more potent antioxidant than R-α-LA. It has been shown to reduce ischemia-reperfusion injury in rat hypoxic hearts and hind limbs^5^ and to protect neurons against ischemic damage by diminishing the accumulation of reactive oxygen species within the cerebral tissue.^5^ Pretreatment with α-LA can protect cultured neurons against injury caused by cyanide, glutamate, or iron ions.^5^ However, it is unknown whether α-LA treatment can counteract hypoxic insults on grafted neural stem cells.

We have long been interested in determining methods to replace cholinergic motor neurons in the spinal cord after spinal cord injury or in motor neuron diseases. Thus, we focused our experiments and discussion in the setting of spinal cord transplantation. In this project, we present an integrated view of needle injection–induced hypoxia-reperfusion and lipid peroxidation damages, which adversely affected the survival of human neural stem cells (hNSCs). By utilizing different detection methods, we revealed the protective effect of LA against lipid peroxidation, which ultimately enhanced the survival of hNSC-derived neurons after transplantation into rat spinal cords.

## Materials and methods

### Cell culture

The established K048 line of hNSCs was previously isolated from the forebrain of a 9-week fetus.^13^ Cells were cultured in 75 cm^2^ flasks as neurospheres. Growth media contained a basic medium consisting of Dulbecco’s Modified Eagle Medium (high glucose, L-glutamine)/Ham’s-F12 (3:1) (Invitrogen/GIBCO, Carlsbad, CA, USA), 15 mM HEPES (4-(2-hydroxyethyl)-1-piperazineethanesulfonic acid, Sigma, St Louis, MO, USA), 1.5% D-glucose (Sigma), 67 μg/mL penicillin/streptomycin (Cellgro, Manassas, VA, USA), and 2 mM L-glutamine (Sigma). The basic media was modified with N-2 Supplement,^14^ which included 25 μg/mL bovine insulin (Sigma), 100 μg/mL human transferrin (Sigma), 100 μM putrescine (Sigma), 20 nM progesterone (Sigma), and 30 nM sodium selenite (Sigma). Growth media was further supplemented with 20 ng/mL recombinant human epidermal growth factor (R&D Systems, Minneapolis, MN, USA), 20 ng/mL recombinant human basic fibroblast growth factor (bFGF) (R&D Systems), 5 μg/mL heparin (Sigma), and 10 ng/mL recombinant human leukemia inhibitory factor (Chemicon, Temecula, CA, USA). Two-thirds of the medium was changed every 3–4 days. Cells were passaged every 10–11 days through mechanical and enzymatic dissociation in 0.025% trypsin (Sigma) plus 0.6% D-glucose (Sigma) in calcium- and magnesium-free Dulbecco’s phosphate buffered saline (CMF-dPBS) (CellGro) for 15 minutes at 37°C according to our previous description.^15^ The reaction was halted with 1.2 mg/mL trypsin inhibitor (Sigma) diluted in conditioned growth media. Cells were incubated at 37°C with 8.5% CO_2_ to maintain a pH of 7.4.

### Cell priming and differentiation

Priming was performed in either 96-well plates, 24-well plates, or 25 cm^2^ flasks that were pretreated with 0.01% poly-D-lysine (Sigma) in CMF-dPBS for 1 hour at 37°C and then coated with 1 μg/cm^2^ laminin (LMN) (GIBCO) in CMF-dPBS overnight at 37°C. Three to 4 days after passage (passage 15–35), hNSCs in neurospheres were resuspended in priming medium and plated in PDL/LMN-coated wells or flasks at a density of 80,000–100,000 cells/cm^2^. Priming media consisted of the basic media and N-2 described above, and was supplemented with 10 ng/mL bFGF, 2.5 μg/mL heparin, and 1 μg/mL LMN. Cells in priming media were incubated at 37°C with 8.5% CO_2_. After 4 days, the priming medium was removed and equal volumes of differentiation medium were added to each well or flask. Differentiation medium consisted of the basic medium described above supplemented with B27 (20 μL/mL) (GIBCO). Cells in differentiation media were incubated at 37°C with 5% CO_2_ to maintain a pH of 7.4.

### Oxidants and antioxidants

LA (a kind gift from GeroNova Research, Inc, Carson City, NV, USA) or vehicle (100% ethanol) was added after 1 day in B27 medium. LA contained a 1:1 mixture of R-α-LA and DHLA. The LA solutions were prepared in 100% ethanol in a nitrogen-filled glove-bag to minimize oxidation of DHLA at a stock concentration of 200 mM LA. An equivalent volume of vehicle was always added to the control wells. After 2 days in B27 medium, cells were subjected to treatment with SIN-1 chloride (Cayman Chemical, Ann Arbor, MI, USA), a peroxynitrite donor, or hydrogen peroxide (H_2_O_2_) (Sigma). SIN-1 and H_2_O_2_ stock solutions were prepared in distilled water (dH_2_O) at various concentrations.

### Hypoxia-reperfusion injury in vitro

Cells in 96-well and 24-well plates at a density of 80,000–100,000 cells/cm^2^ were incubated at 37°C in a 3% oxygen chamber for 24 hours to mimic a hypoxic environment. To imitate reperfusion, they were then immediately incubated at normoxia at 37°C for 1 or 4 hours prior to performing lactate dehydrogenase (LDH) or water-soluble tetrazolium-1 (WST-1) cell viability assays.

### TUNEL staining

After 2 days in B27 medium, hNSCs in 24-well plates at a density of 80,000–100,000 cells/cm^2^ were fixed in ice-cold 4% paraformaldehyde (PFA) for 20 minutes. For TUNEL (terminal-deoxynucleotidyl-transferase-mediated dUTP nick end labeling) staining, the ApopTag® Fluorescein In Situ Apoptosis Detection Kit S7110 (Chemicon) was used according to the manufacturer’s instructions. Counterstaining with DAPI (4′,6-diamidino-2-phenylindole) (1:1,000) was performed, and coverslips were mounted with Fluoromount-G. Images were acquired with a Nikon 80i epifluorescent microscope (Nikon, Tokyo, Japan) using the NIS-Elements imaging software.

### LDH assay

Cell death was detected using the Cytotoxicity Detection Kit (Roche, Indianapolis, IN, USA) according to the manufacturer’s protocol. Briefly, 350 μL of conditioned medium from each sample in 24-well plates was collected and immediately placed on ice in sterile microcentrifuge tubes. Samples were centrifuged at 14,000*g* for 2 minutes to remove cellular debris and the supernatant was transferred to new tubes. After vortexing, 100 μL of medium from each sample was plated in triplicate in 96-well plates. 100 μL of LDH reagent was added to each well. Plates were incubated at room temperature in the dark for 30 minutes. The absorbance was measured at a wavelength of 490 nm with a 630 nm reference using an ELx800UV Universal Microplate Reader (BioTek, Winooski, VT, USA).

### WST-1 assay

Cell viability was assessed using the WST-1 kit (Roche). Mitochondrial dehydrogenases in live cells cleave WST-1, a tetrazolium salt, into a colorimetric product, formazan, which was assayed to determine the amount of viable cells. The assay was performed according to the manufacturer’s instructions using samples in 96-well plates. A 1:10 dilution of the WST-1 reagent was incubated with the cells for 1.5 hours. The absorbance of the formazan produced by metabolically active cells in each sample was measured at a wavelength of 450 nm with a 630 nm reference using an ELx800UV Universal Microplate Reader.

### Activated caspase 3 assay

Caspase 3 activity was detected using the EnzChek® Kit (Molecular Probes, Eugene, OR, USA) according to the manufacturer’s instructions. Briefly, 20 μg of protein lysates, collected from 25 cm^2^ flasks, were pipetted in triplicates into 96-well plates (N = 3/group), dithiothreitol (DTT) (5 mM) and Rhodamine 110, bis-(N-acetyl-L-aspartyl-L-glutamyl-L-valyl-l-aspartic acid amide)(Z-DEVD-R110) (25 μM) were added to each well. Z-DEVD-R110 contains the specific caspase 3 cleavage site DEVD. Active caspase 3 cleaves DEVD releasing the R110 fluorescent tag. Plates were read in a fluorescent plate reader at 490 nm excitation and 520 nm emission, once per minute for 1 hour. A linear regression of fluorescence/μg protein/minute was generated. The slope of the line represented caspase 3 activities. Values for the triplicates of each sample were averaged to obtain the final slope of each sample. Each group contained three samples. The average slopes from the three samples within each group were compared.

### Bromodeoxyuridine staining

For bromodeoxyuridine (BrdU) labeling, 1 μM BrdU (BD Biosciences, San Jose, CA, USA) was added after 1 day in B27 medium, simultaneously with LA administration. For immunodetection of BrdU incorporation, 48 hours after labeling, cells were fixed in ice-cold 4% PFA for 20 minutes at room temperature followed by a second fixation in ice-cold 100% methanol for 20 minutes at −20°C. Cells were then permeabilized in 2N hydrochloric acid for 20 minutes at 37°C. The acid was neutralized with borate buffer (pH 8.5). Cells were then blocked in 1% BSA–0.5% Tween 20 (Sigma) and 5% normal goat serum for 1 hour at room temperature. A monoclonal antiBrdU antibody (1:1000) (Sigma) was added for 1 hour at room temperature. An Alexa Fluor® 568-conjugated goat antimouse secondary antibody (1:300) (Molecular Probes) was added for 2 hours at room temperature in the dark. Counterstaining with DAPI (1:1,000) was performed and coverslips were mounted with Fluoromount-G. Images were acquired with a Nikon 80i epifluorescent microscope using the NIS-Elements imaging software. In all experiments, cells were stained with secondary antibody only, and cells that did not receive BrdU pulse labeling served as negative controls.

### Western blot analysis

Western blot analysis was performed as described previously.^15,16^ One day after differentiation, cells were treated with vehicle (ethanol), 0.25 mM LA, or 10 ng/mL insulin-like growth factor 1 (IGF-1) (R&D Systems Inc). After 24 hours, cells were washed in CMF-dPBS and lysed with Cell Lysis Buffer (Cell Signaling Technology, Danvers, MA, USA) supplemented with phenylmethylsulfonyl fluoride (Cell Signaling Technology) in Molecular Grade Water (Cellgro). Lysates were collected from 25 cm^2^ flasks with a cell scraper and transferred to microcentrifuge tubes. Samples were mixed at 4°C for 1 hour. Centrifugation was performed at 14,000*g* at 4°C for 10 minutes and the supernatant was transferred to new tubes. Protein lysates were quantified with the bicinchoninic acid protein quantification kit (Pierce Biotechnology, Rockford, IL, USA) and stored at −80°C. Protein (30 μg) was diluted in NuPAGE® LDS sample buffer and reducing agent (Invitrogen) and heated to 70°C for 10 minutes. Samples were loaded into 4%–12% NuPAGE® Novex Bis Tris Gels (Invitrogen), and electrophoresis was performed at 150 V for approximately 2 hours. Gels were transferred onto Hybond ECL nitrocellulose membrane (GE Healthcare, Little Chalfont, Buckinghamshire, UK) by electrophoretic transfer at 30 V for 1.5 hours at 4°C. Membranes were blocked in 5% nonfat milk in 0.1% Tween 20 (Sigma) in Tris-buffered saline (0.01M Tris base mixed with 0.1M sodium chloride, both from Sigma)(0.1% Tween-TBS) for 1 hour at room temperature. Primary antibodies, including rabbit polyclonal antiAkt (1:1,000) (Cell Signaling Technology) and rabbit polyclonal antiphospho-Akt (1:1,000) (Cell Signaling Technology ), were diluted in 5% nonfat milk in 0.1% Tween-TBS and added to the membrane overnight at 4°C. Membranes were washed with 0.1% Tween-TBS and incubated for 1 hour at room temperature with donkey antirabbit horseradish peroxidase-conjugated secondary antibody (1:7,500) (Amersham Biosciences). Membranes were washed and subjected to an ECL Western Blot Detection System (Amersham Biosciences) and ECL hyperfilm (Amersham Biosciences). Membranes were stripped for 30 minutes with Restore Western Blot Stripping Buffer (Pierce Biotechnology) and reprobed for the loading control mouse anti-β-actin (1:25,000) (Sigma) and sheep antimouse horseradish peroxidase-conjugated secondary antibody (1:7,500) by the same protocol.

### Glutathione assay

Reduced glutathione was detected using the Glutathione (GSH) Detection Kit (Chemicon) according to the manufacturer’s instructions. Briefly, 20 μg of protein lysates, collected from 25 cm^2^ flasks, were pipetted in triplicates into 96-well plates. Monochlorobimane was added to each well and plates were incubated at room temperature in the dark for 2 hours. Plates were read in a fluorescence plate reader at 380 nm excitation and 460 nm emission.

### Transplantation

All surgical protocols were established according to the National Institutes of Health guidelines for the care and use of laboratory animals and approved by the University of Texas Medical Branch Institutional Animal Care and Use Committee. hNSCs were cultured in growth media and priming media as described above. However, half the priming medium was changed after 2 days. On day 3 in priming medium, hNSCs were transduced by a recombinant adeno-associated viral vector (AAV) containing the enhanced green fluorescent protein gene (egfp), AAVegfp, according to our previous description.^17,18^ Cells were then cultured in B27 differentiation medium after a 5-day priming period. Either 0.25 mM LA or an equivalent volume of ethanol was added to the cells after 1 day in B27 differentiation medium. Twenty-four hours later, cells were dissociated and resuspended in fresh B27 medium containing 3 nM FK506 (Tacrolimus) (Alexis Biochemicals, Plymouth Meeting, PA, USA) and 250 U/mL DNase (Sigma). Either 0.25 mM LA or ethanol was also added and the cells were stored on ice during the transplantation process. Two month old Sprague-Dawley male rats received a stereotactic transplantation of hNSCs using a 5 μL Hamilton syringe with a 26-gauge needle according to our previously described protocol.^17,18^ Approximately 1 × 10^5^ hNSCs in a total volume of 2 μL were injected into the ventral horns of spinal cords unilaterally at L4-L5. Three rats were transplanted in each group. All transplanted rats were immunosuppressed with NEORAL cyclosporine (Novartis Pharmaceuticals, East Hanover, NJ, USA) at 100 μg/mL in drinking water beginning 3 days prior to transplantation surgery and then throughout their lifespan.

### Detection of tissue hypoxia

Briefly, rats were anesthetized with pentobarbital and transcardially perfused with 0.1 M PBS followed by 4% PFA. Spinal cords were dissected and cord sections were postfixed in 4% PFA at 4°C overnight, washed in PBS, and incubated in 30% sucrose at 4°C for 48 hours. Sections were then embedded in Tissue-Tek® OCT Compound (Sakura Finetek Europe B.V., Flemingweg, The Netherlands), sectioned at 40 μm on a cryostat and stored at −20°C. The Hypoxyprobe™-1 Kit (Chemicon) was used for the detection of tissue hypoxia; ie, below a pO_2_ of 10 mmHg, according to the manufacturer’s protocol. Images were acquired with a Nikon (Nikon, Tokyo, Japan), D-Eclipse C1 Laser Scanning Confocal microscope.

### 4-Hydroxynonenal immunostaining

Cells were fixed in 4% PFA for 20 minutes at room temperature, washed with PBS, and postfixed with 0.3% H_2_O_2_ in methanol for 30 minutes at room temperature. Tissue sections were prepared as described above. The Vectastain ABC kit (Vector Laboratories, Burlingame, CA, USA) was used for staining of 4-hydroxynonenal (4-HNE) Michael adducts. Cells or tissue sections were blocked in 1.5% normal serum and incubated with primary rabbit polyclonal anti-4-HNE (1:3,000) (EMD Millipore, Billerica, MA, USA) overnight at 4°C, followed by incubation with Goat antirabbit IgG (1:200) (Vector Laboratories) for 30 minutes at room temperature. After treatment with the avidin-biotin-peroxidase complex for 30 minutes, cell or tissue samples were incubated with 0.025% diaminobenzidine tetrahydrochloride (DAB) (Sigma) and 0.01% H_2_O_2_ for 6 minutes at room temperature. This is followed by rinsing with water and dehydrating with 50%, 70%, 95%, and 100% ethanol. Coverslips were dipped in xylene and mounted with Permount. Images were taken by Nikon light microscopy.

### Stereology

One month after transplantation, the L4/L5 grafting site sections were dissected and serially sectioned at 40 μm using a cryostat. Green fluorescent protein (GFP)^+^-grafted hNSCs in the ventral horn were determined stereologically using a fractionator analysis ^19^ on a Nikon 80i epifluorescent microscope.

### Statistics

Statistical analyses were done using the GraphPad Prism Version 4 software (GraphPad Software, San Diego, CA, USA). The Student’s *t*-test was used when comparing two groups. A one-way analysis of variance (ANOVA) with Dunnett’s posttest was used for comparing multiple groups. A two-way ANOVA with Bonferroni posttests was used to analyze data sets with two variables. A *P* value less than 0.05 was considered statistically significant. All data were expressed as mean ± standard error.

## Results

### Transplantation needle adversely affects hNSC survival

**Figure 1:**
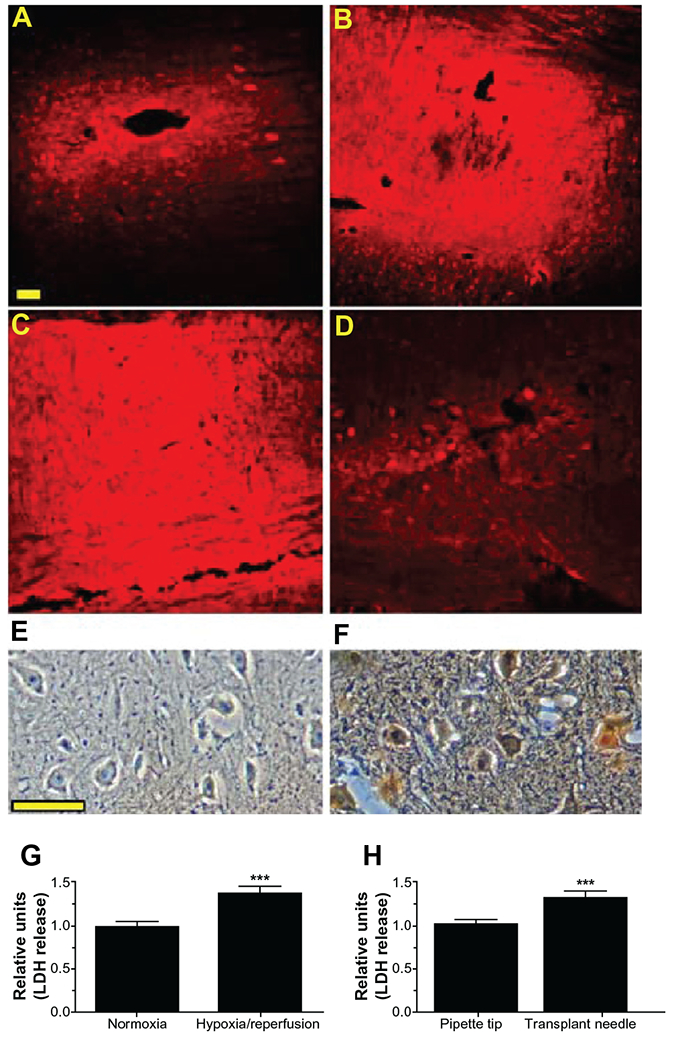
Transplantation needle adversely affects hNSC survival. **Notes:** Representative images of the spinal cord at the needle injection site (**A**) 30 minutes, (**B**) 1 hour, (**C**) 12 hours, and (**D**) 24 hours after needle injection. Hypoxyprobe™-1 binds the hypoxic cells that contain a pO_2_ level less than 10 mmHg at 37°C. Images labeled by 4-HNE adduct (brown stain), a lipid peroxidation marker, (**E**) before and (**F**) 48 hours after needle injection. Scale bars = 100 μm. (**G**) LDH release from hNSCs treated for 24 hours under 3% oxygen hypoxia followed by a I-hour reperfusion at normoxia. N = 5/group. (**H**) LDH release from hNSCs 24 hours after passing through a transplantation needle at a rate of 0.2 μL/minute or a plastic pipette tip. N = 3/group. *P* < 0.001. Data analyzed by Student’s *t*-test. **Abbreviations:** 4-HNE, 4-hydroxynonenal; hNSC, human neural stem cells; LDH, lactate dehydrogenase; pO_2_, partial pressure of oxygen.

Several steps of the cell transplantation process may ultimately affect the immediate survival of grafted cells. We first assessed to what extent injection per se creates a hypoxic environment into which hNSCs are deployed. A transplantation needle was inserted into the lumbar spinal cords of 12 Sprague-Dawley rats at 2 months of age. Transplantation medium without cells was injected into the cords. Three rats each were sacrificed at 30 minutes, 1 hour, 12 hours, and 24 hours, and the spinal cord tissue was collected. The Hypoxyprobe™-1 was used for detection of hypoxic cells (pO_2_ less than 10 mmHg) in the cord tissue around the site of needle insertion. Representative images of hypoxia detection are shown at 30 minutes (Figure 1A), 1 hour (Figure 1B), 12 hours (Figure 1C), and 24 hours (Figure 1D). The red fluorescent hypoxic tissue was initially found closely around the needle injection site at 30 minutes. The area of tissues undergoing hypoxic attack then extended away from the transplantation site, and reached a peak level between 1 and 12 hours. At 24 hours, the pO_2_ began returning to levels greater than 10 mmHg, which indicated reperfusion of the tissue. Intact spinal cord tissues taken from control rats showed no signs of lipid peroxidation (Figure 1E), as indicated by the lack of 4-HNE immunoreactivity. However, lipid peroxidation damage (brown staining) was present (Figure 1F) 48 hours after needle insertion into the spinal cord, indicating an increase in free radical production upon reperfusion.

To determine the potential effects of hypoxia-reperfusion injury on hNSC survival during transplantation, primed and differentiated hNSCs, similar to those used in transplantation, were incubated in an oxygen chamber containing 3% oxygen (hypoxia), or an approximate pO_2_ of 23 mmHg, for 24 hours. Normal oxygen levels at sea level are 21% oxygen, which corresponds to a pO_2_ of 160 mmHg. An oxygen level of 3% closely corresponds to the hypoxic environment created in the cord tissue around the site of needle insertion. After a 1-hour reperfusion at normoxia, there was a significant increase (39%) in the amount of LDH released into the medium as compared to cells that were not exposed to hypoxic conditions (Figure 1G).

In addition to creating a hypoxic environment at the insertion site, the use of the transplantation needle may directly harm cells. Differentiated hNSCs were injected into culture plates using either a transplantation needle or a p200 pipette. The injection rate was kept at 0.2 μL/minute, similar to that of transplantation into spinal cords. The LDH levels were significantly higher (30%) in cells 24 hours after injection through the needle when compared to those using the pipette (Figure 1H).

### LA enhances hNSC survival in vitro

**Figure 2:**
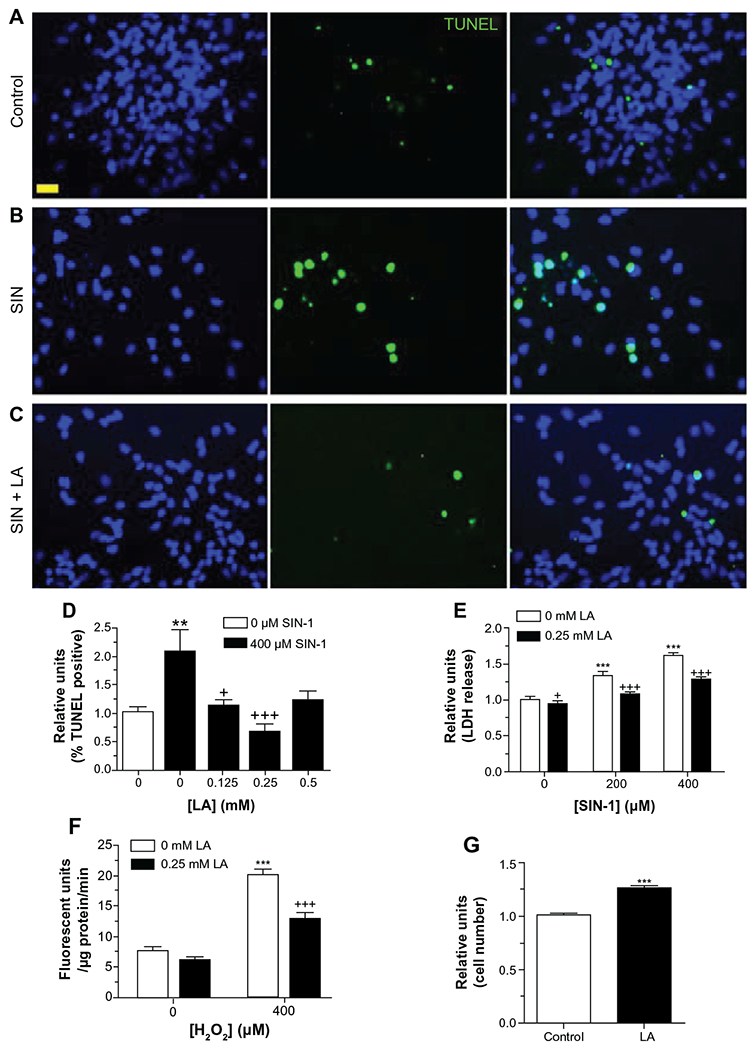
Lipoic acid promotes hNSC survival in vitro. **Notes:** Representative images of TUNEL-positive hNSCs 24 hours after treatment with (**A**) vehicle (dH_2_O and ethanol), (**B**) 400 μM SIN-1 and vehicle (ethanol), and (**C**) 400 μM SIN-1 following a 24-hour preincubation with 0.25 mM LA. DAPI nuclear counterstain in blue, TUNEL in green, and merged images are shown. Scale bar = 20 μm. (**D**) Cumulative TUNEL labeling in hNSCs treated with a 24-hour preincubation with LA followed by SIN-1 for 24 hours. Data normalized to the 0 mM LA/0 μM SIN-1 control group (white bar). N = 3/group. **P < 0.05 compared to 0 mM LA/0 μM SIN-1 control. ^+^*P* < 0.05 ^+++^P < 0.001 compared to 0 mM LA/400 μM SIN-1. Data analyzed by Student’s *t*-test (**E**) LDH release from 0.25 mM LA-pretreated hNSCs 24 hours after exposure to SIN-1. Data normalized to 0 μM SIN-1/0 mM LA control. N = 8/group. ***P < 0.001, compared to 0 μM SIN-1/0 mM LA control. ^+++^*P* < 0.001, compared to associated white bars. Data analyzed by two-way ANOVA with Bonferroni posttests. Serial SIN-1 concentrations in 0 mM LA groups analyzed by one-way ANOVA with Dunnett’s posttest (**F**) Caspase 3 activity in hNSCs 24 hours after treatment with H_2_O_2_, which followed a 24-hour preincubation period with 0.25 mM LA. N = 3/group. ***P < 0.001, compared to 0 μM H_2_O_2_/0 mM LA control. ^+++^*P* < 0.001, compared to associated white bar. Data analyzed by two-way ANOVA with Bonferroni posttests. 0 μM H_2_O_2_/0 mM LA and 400 μM H_2_O_2_/0 mM LA compared by Student’s *t*-test (**G**) WST-1 cell viability assay of hNSCs exposed to hypoxia (3% oxygen) for 24 hours followed by a 4-hour reperfusion at normoxia. Cells were treated with vehicle (ethanol) or 0.25 mM LA for 24 hours prior to hypoxia. Data normalized to the vehicle control group. N = 12/group. ***P < 0.001. Data analyzed by Student’s *t*-test. **Abbreviations:** ANOVA, analysis of variance; DAPI, 4′,6-diamidino-2-phenylindole; hNSCs, human neural stem cells; LA, lipoic acid; min, minute; TUNEL, terminal-deoxynucleotidyl-transferase-mediated dUTP nick end labeling; WST, water-soluble tetrazolium.

Upon reperfusion to the hypoxic area, there is an increase in free radical formation and lipid peroxidation damage from peroxynitrite. To determine whether peroxynitrite was detrimental to hNSC survival, differentiated hNSCs were exposed to SIN-1, a peroxynitrite donor, for 24 hours. A broad spectrum free radical scavenger, LA, was preincubated with the hNSCs for 24 hours prior to SIN-1 addition. Cell death was determined by the number of TUNEL-positive cells. Representative images of TUNEL staining is shown in differentiated hNSCs exposed to vehicle (ethanol and dH_2_O) only (Figure 2A), vehicle (ethanol) plus 400 μM SIN-1 (Figure 2B), and 0.25 mM LA plus 400 μM SIN-1 (Figure 2C). SIN-1 dramatically decreased the total cell number and increased the number of TUNEL-positive cells (Figure 2B). Preincubation with 0.25 mM LA for 24 hours negated hNSC loss and decreased the number of TUNEL-positive cells (Figure 2C). Cumulative results with serial concentrations of LA are shown in Figure 2D. Treatment with 400 μM SIN-1 resulted in a significant increase in the percentage of TUNEL-positive cells (107%). Preincubation with LA ameliorated hNSC loss in the presence of SIN-1, causing significant decreases in TUNEL-positive cells (0.125 mM, 46% reduction; 0.25 mM, 67% reduction; 0.5 mM, 41.5% reduction). Although each tested LA concentration protected hNSCs from SIN-1-mediated death, 0.25 mM LA showed the most significant protection and was used throughout the remainder of the experiments.

Additional data, utilizing different detection methods for cell death, also revealed the protective effect of LA for hNSCs against oxidative stress. A peroxynitrite donor, SIN-1, caused significant increases in hNSC death after 24 hours at 200 μM (34% increase) and 400 μM (62% increase) as determined by LDH release (Figure 2E). When hNSCs were pretreated with 0.25 mM LA for 24 hours, LDH release significantly declined in groups treated with and without SIN-1 (0 μM SIN-1, 7% decrease; 200 μM SIN-1, 20% decrease; 400 μM SIN-1, 21% decrease).

Hydrogen peroxide also caused hNSC death, and increased the level of activated caspase 3 by 1.6-fold in hNSCs at 24-hours after exposure to 400 μM H_2_O_2_ (Figure 2F). Treatment with 0.25 mM LA for 24 hours prior to the addition of H_2_O_2_ partially protected hNSCs as indicated by a 36% decrease in caspase 3 activity.

Since reactive oxygen species are produced and damage cells upon reperfusion to a hypoxic area, we also determined whether preincubation with LA could protect hNSCs from hypoxia-reperfusion injury. hNSCs were exposed to hypoxia (3% oxygen) for 24 hours followed by a 4-hour reperfusion at normoxia. Pretreatment with 0.25 mM LA for 24 hours prior to hypoxia-reperfusion significantly improve the survival of hNSCs by 26% as determined using the WST-1 cell viability assay (Figure 2G).

### LA increases glutathione and prevents lipid peroxidation

**Figure 3:**
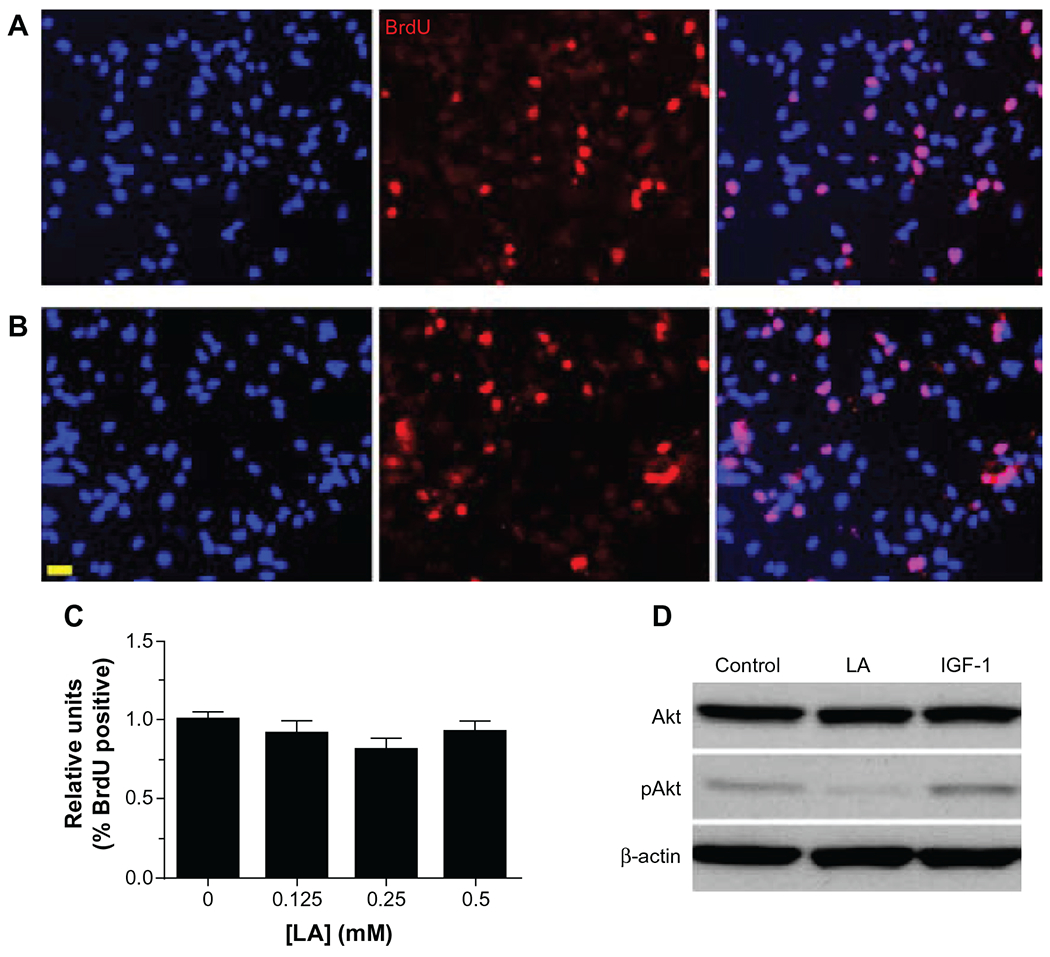
Effects of lipoic acid on hNSC proliferation and Akt signaling. **Notes:** Representative images of BrdU-labeled hNSCs treated with (**A**) vehicle (dH_2_O) and (**B**) 0.25 mM LA. DAPI in blue, BrdU in red, and merged images are shown. Scale bar = 20 μm. (**C**) Cumulative BrdU results of hNSCs 48 hours after the addition of BrdU and LA. Data normalized to the vehicle control group and analyzed by one-way ANOVA. N = 3/group. (**D**) Western blot analyses of Akt activation/phosphorylation in hNSCs 24 hours after the addition of vehicle (ethanol), 0.25 mM LA, and 10 ng/mL IGF-1. β-actin serves as internal control. **Abbreviations:** ANOVA, analysis of variance; BrdU, bromodeoxyuridine; DAPI, 4′,6-diamidino-2-phenylindole; hNSCs, human neural stem cells; IGF-1, insulin-like growth factor 1; LA, lipoic acid.

To determine the mechanism through which LA protects hNSCs from oxidative stress-mediated cell death, we first applied the BrdU incorporation technique to test whether LA increased cell numbers by stimulating cell division. Representative images of BrdU-labeled cells 48 hours after treatment with BrdU, together with vehicle (ethanol) or 0.25 mM LA, are shown in Figure 3A and B, respectively. Cumulative results with serial concentrations of LA are shown in Figure 3C, which are translated to 21%–25% of cells labeled with BrdU during the 48-hour period of time. These data indicated that LA treatment did not significantly affect hNSC proliferation.

We then determined whether LA conferred protection to hNSCs through the activation of Akt, a pro-survival signaling molecule. One day after differentiation, hNSCs were treated with vehicle (ethanol), 0.25 mM LA, or 10 ng/mL IGF-1. Western blot analyses showed that IGF-1, as expected, increased the phosphorylation of Akt, whereas LA appeared to decrease Akt phosphorylation 24 hours after treatment (Figure 3D).

**Figure 4:**
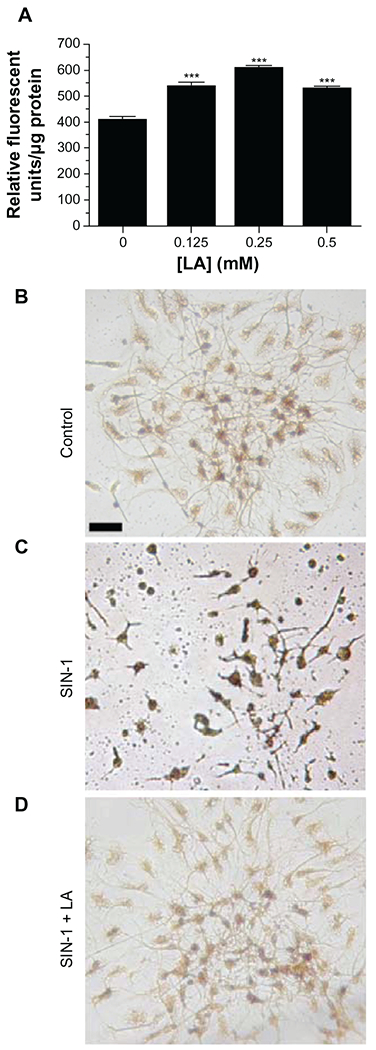
Lipoic acid increases reduced glutathione and prevents lipid peroxidation. **Notes:** (**A**) Levels of reduced GSH in hNSCs 24 hours after the addition of vehicle (ethanol) or serial concentrations of LA. N = 6/group. ***P < 0.001, compared to 0 mM LA. Data analyzed by one-way ANOVA with Dunnett’s posttest. (**B-D**) Representative images of the 4-HNE adduct (brown stain), a lipid peroxidation marker, in hNSCs treated by (**B**) vehicle (dH_2_O and ethanol), (**C**) a 24-hour preincubation with vehicle (ethanol) followed by 24 hours of 400 μM SIN-1, and (**D**) a 24-hour preincubation with 0.25 mM LA followed by 24 hours of SIN-1. Scale bar = 50 μm. **Abbreviations:** 4-HNE, 4-hydroxynonenal; ANOVA, analysis of variance; GSH, glutathione; hNSCs, human neural stem cells; LA, lipoic acid.

LA is a powerful reducing agent known to increase the pool of an intracellular potent antioxidant, reduced GSH. One day after differentiation, hNSCs were treated with vehicle or serial concentrations of LA for 24 hours. Cell lysates were analyzed for the relative amount of GSH using monochlorobimane, which produces fluorescence upon binding to GSH. Each concentration of LA significantly increased the amount of GSH compared to the control group, as shown in Figure 4A.

Finally, LA has been shown to act as a broad spectrum antioxidant. Thus, we determined whether LA could prevent damage by lipid peroxidation to hNSCs. One day after differentiation, hNSCs were treated with either vehicle or 0.25 mM LA. After 24 hours, SIN-1, a peroxynitrite donor, was added to the cells at a 400 μM concentration. Immunocytochemistry was used to detect the amount of 4-HNE damage, a marker of lipid peroxidation. Cells that underwent lipid peroxidation appear brown. Figure 4B shows a representative image of 4-HNE staining in hNSCs treated with vehicle (ethanol and dH_2_O). SIN-1 dramatically increased 4-HNE staining (Figure 4C), whereas pretreatment with LA reduces the amount of 4-HNE damage in the presence of SIN-1 to levels similar to the control group (Figure 4D).

### LA enhances transplanted hNSC survival

**Figure 5:**
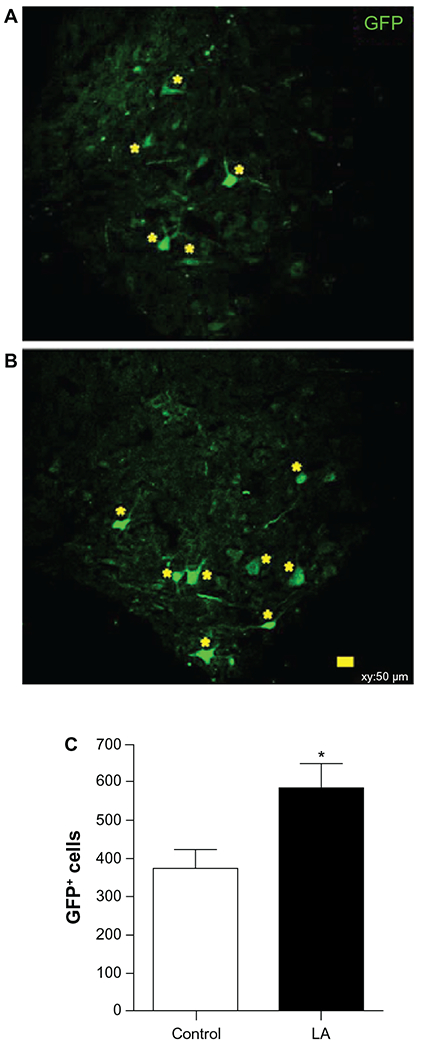
Lipoic acid increases transplanted hNSC survival in vivo. **Notes:** Representative images of transplanted GFP-labeled hNSCs treated with (**A**) vehicle (ethanol) and (**B**) 0.25 mM LA. Scale bar = 50 μm. Yellow stars indicate GFP-labeled grafted cells. (**C)** Cumulative cell survival of grafted hNSCs pretreated with either vehicle (ethanol) or 0.25 mM LA. N = 3/group. *P < 0.05. Data analyzed by Student’s *t*-test. **Abbreviations:** GFP, green fluorescent protein; hNSCs, human neural stem cells; LA, lipoic acid.

In order to determine whether pretreatment with LA increases the survival of transplanted cells, one day-differentiated hNSCs were treated with either vehicle (ethanol) or 0.25 mM LA for 24 hours prior to transplantation. Three Sprague-Dawley male rats, age 2 months, received stereotactic spinal transplantation of hNSCs from each treatment group unilaterally at L4/L5. One month after transplantation, stereological analysis was performed at the grafting sites of each rat. Many of GFP-labeled cells (represented by asterisks) acquired a neuronal morphology in the ventral horn of the rat spinal cord (Figure 5) as we previously demonstrated.^18^ Some GFP-labeled cells appeared to be motor neurons, based on the characteristic size and morphology, but further confirmation of the phenotype is needed with immunohistochemical analysis. Compared to the control group (Figure 5A), LA pretreatment significantly increased the total number of GFP^+^ cells by 58% (average 583 ± 64.4 versus 370 ± 51.5 per rat) (Figure 5B and C), indicating an enhancement of transplanted cell survival.

## Discussion

The major focus of the present study was to determine whether LA, a natural antioxidant, could enhance the survival of human neural stem cells in the setting of hypoxia-reperfusion and oxidative stress, both of which may mediate transplantation-associated cell graft injury. We first demonstrated that the needle injection per se could be injurious to hNSCs during the transplantation process by inducing hypoxia-reperfusion injury at the graft site. We further revealed that LA could protect hNSCs from the consequential hypoxia-reperfusion injury and oxidative stress-mediated cell death.

Much of our knowledge on neural transplantation has been bruit on the pioneer studies over the past four decades, primarily by using the embryonic neurons.^20^ One of the key issues in this field is the survival of the grafted cells, which is affected by many factors, including intrinsic properties of the donor cells and extrinsic factors from the grafting procedure and the host.^21^ For the latter, anoxia, hypoglycemia, excitotoxicity, inflammation, oxidative stress, and proteolysis have all been suggested to contribute to the death of implanted neurons.^22,23^ Here, we focus on the mechanical trauma associated with the needle injection, which has been known to interrupt normal vasculature and subsequently cause anoxia and/or hypoxia. However, it was unclear how long a hypoxic environment ensues following a needle insertion. Using the new and sensitive Hypoxyprobe, we found that a needle injection of 2 μL solution did result in local hypoxia, which gradually increased and peaked around 12 hours, and then significantly reduced at 24 hours after injection. The latter indicates reperfusion following the initial hypoxia. Such a hypoxia-reperfusion injury following needle injection may cause oxidative stress to the host and grafted cells in addition to metabolic challenges. Although oxidative stress can be induced directly by hypoxia due to increased cytoplasmic Ca^2+^,^24,25^ subsequent reperfusion with reoxygenation induces a large burst of oxidant production in various tissues.^2,26,27^ Indeed, we found that spinal cord neurons near the injection site underwent oxidative stress by showing strong lipid peroxidation at 48 hours after the needle injection. Furthermore, our in vitro model of hypoxia followed by reperfusion also confirmed a cytotoxic effect on primed and differentiated hNSCs. Protecting cells within the first 48 hours after transplantation, when hypoxia-reperfusion injury peaks, would thus be imperative in improving long-term cell survival and potentially functional outcome.

An antioxidative strategy may enhance the survival of grafted hNSCs. LA has several properties that make it a strong candidate for protecting stem cell transplants from hypoxia-reperfusion injury. At pharmacologic doses, unbound LA directly scavenges a broad-spectrum of reactive species, including superoxide, hydroxyl radical, nitric oxide, and peroxynitrite.^5^ Furthermore, LA can regenerate other endogenous antioxidants, such as reduced glutathione and vitamin E. Maintaining high levels of reduced glutathione may be beneficial in hypoxia-reperfusion injury, since augmented production of oxidative stress is evident upon reperfusion as revealed by increased lipid peroxidation. Finally, LA may protect cells via the activation of the Akt cell survival pathway as well as decreasing inflammation through the NFκB (nuclear factor kappa-light-chain-enhancer of activated B cells) activity.^5^ In summary, LA acts through a variety of mechanisms, which may be particularly useful in protecting grafted stem cells from oxidative stress and hypoxia-reperfusion injury. Our data indicate that LA likely protects hNSCs by enhancing glutathione production, ameliorating lipid peroxidation and reducing apoptotic cell death without activating the Akt survival pathway or increasing cell proliferation. Further work is needed to determine the significance of the apparent decrease in pAKT after treatment with LA. In addition, it is unknown whether and to what extent LA affects human neural stem cell differentiation. Studies will be needed to delineate the differentiation patterns in the presence of LA and to determine which cell types are ultimately protected by LA in the setting of hypoxia-reperfusion injury or direct oxidative stress.

In order to enhance long-term stem cell survival, several types of therapies may ultimately be required to effectively combat several cell death mechanisms induced by hypoxia-reperfusion insults. Several groups have attempted to protect grafted stem cells from the ischemic environment in rodent stroke models using hypoxic preconditioning. Pretreating embryonic stem cell-derived neural progenitor cells in hypoxic conditions (1% oxygen) in vitro promoted increased cell survival 3 days after transplantation in the ischemic rat brain and improved sensorimotor function up to 35 days after transplant compared to cell transplants without hypoxic preconditioning.^28^ Similarly, Francis and Wei reported that human embryonic stem cell-derived neural progenitors that underwent hypoxic preconditioning in 0.1% oxygen for 12 hours prior to differentiation at normoxia and exposure to hydrogen peroxide or oxygen-glucose deprivation showed an approximate 50% increase in cell survival.^29^ This neuroprotection was correlated with significant upregulation in hypoxia-inducible factor (HIF-1α and HIF-2α) as well as their downstream target genes during the hypoxic preconditioning phase. Combining hypoxic preconditioning with LA treatment may counteract different cell death mechanisms during hypoxia-reperfusion, potentially providing a more effective method of optimizing cell transplantation therapy and ultimately improving functional outcome.
